# Spontaneous massive hemothorax as a complication of necrotizing pneumonia in a patient with severe acute respiratory syndrome coronavirus 2 induced acute respiratory distress syndrome: a case report

**DOI:** 10.1186/s13256-021-03032-9

**Published:** 2021-09-03

**Authors:** Carolin Jung, Hans-Joerg Gillmann, Thomas Stueber, Lukas Hinken

**Affiliations:** Department of Anaesthesiology and Intensive Care Medicine, Medical School Hannover, Carl-Neuberg-Str. 1, 30625 Hannover, Germany

**Keywords:** COVID-19, Necrotizing pneumonia, Hemothorax, ARDS, Case report

## Abstract

**Background:**

We present an unusual bleeding complication in a patient with severe acute respiratory distress syndrome in coronavirus disease 2019.

**Case presentation:**

The patient, a 63-year-old Caucasian man, received venovenous extracorporeal membrane oxygenation support after rapid deterioration of lung function on day 6 after admission to hospital. After initial stabilization on lung protective ventilation and prone positioning, he started to develop mild bleeding complications until he went into occult profound hemorrhagic shock. Causative was a massive hemothorax of the right hemithorax with mediastinal shifting due to spontaneous bleeding from a pulmonal artery in a heavily remodeled right inferior lobe. Histopathological examination of the resected tissue showed signs of an organizing fibrinous pneumonia with focal parenchyma necrosis. After surviving a massive bleeding event caused by necrotizing pneumonia, the patient made a swift recovery and was discharged to rehabilitation 31 days after initial hospital admission.

**Conclusions:**

The combination of severely elevated inflammatory markers and pulmonary hemorrhage should arouse suspicion of necrotizing pneumonia. In necrotizing pneumonia, the possibility of severe intrathoracic bleeding complications should be kept in mind if it comes to sudden deterioration of the patient.

**Supplementary Information:**

The online version contains supplementary material available at 10.1186/s13256-021-03032-9.

## Background

Pulmonary hemorrhage is a recurring finding in patients with severe acute respiratory syndrome coronavirus 2 (SARS-CoV-2) infections and has been reported in 17% of cases with severe coronavirus disease 2019 (COVID-19) on extracorporeal support [1]. Necrotizing pneumonia is strongly associated with the occurrence of pulmonary hemorrhage and severely elevated inflammatory markers and comes with a poor prognosis [2, 3]. It can induce profound parenchyma damage and lead to severe complications such as spontaneous hemothorax. Awareness of the possibility of this complication can lead to timely diagnostic and therapy.

We report a case of spontaneous hemothorax as a complication of necrotizing pneumonia in a patient with severe COVID-19, that took a favorable course after surgical resection of the necrotic tissue.

## Case presentation

A 63-year-old Caucasian male with a background of arterial hypertension and mild obesity (BMI 30.5 kg/m^2^) was admitted to hospital with dyspnea under COVID-19. He initially tested positive for SARS-CoV-2 via a polymerase chain reaction swab conducted in the ambulatory setting. When he arrived at the primary care hospital, he displayed a reduced general condition. Admission observations revealed: blood pressure 100/40 mmHg, heart rate 115 beats/minute, peripheral oxygen saturation 71%, body temperature 40 °C. His breathing noises were reduced, and there were rattling noises over his lungs bilaterally. He was cooperative and orientated, and showed no focal neurological deficit. His renal function markers were elevated [creatinine 1.66 mg/dl, estimated glomerular filtration rate (eGFR) 45 ml/minute], and the liver function markers were in the normal range. He was admitted to intensive care unit (ICU) 5 days later owing to worsening of respiratory symptoms. After 1 day of noninvasive ventilation, the patient decompensated into global respiratory insufficiency, and endotracheal intubation was required. Because of persistent global respiratory insufficiency under exhausted conservative therapy, the patient was transferred to our university hospital for venovenous extracorporeal support (vvECMO) in severe acute respiratory distress syndrome (ARDS). Computed tomographic (CT) imaging before transferal showed diffuse bilateral ground-glass opacities. On admission at our hospital, his acute phase reactants were elevated: white blood cell count 20,400/µl (normal range 3600–10,000/µl), CRP 316.5 mg/l (normal range < 5 mg/l), IL-6 420 ng/l (normal range < 7 ng/l), soluble IL-2-receptor 1549 kU/l (normal range 223–710 kU/l), ferritin 916 µg/l (normal range 27–365 µg/l), and fibrinogen > 9 g/l (normal range 1.8–3.5 g/l). He showed lymphocytopenia with a cell count of 470/µl (2.3%). SARS-CoV-2-PCR diagnostic was repeated from tracheal aspirate on arrival at our hospital and remained positive (cycle threshold 25). His medical treatment taken at home included valsartan, bisoprolol, amlodipine, and chlorthalidone as well as atorvastatin and mirtazapine. The patient did not smoke or consume alcohol regularly. He is married and has been working as a specialist in internal medicine until his recent retirement.

**Fig. 1:**
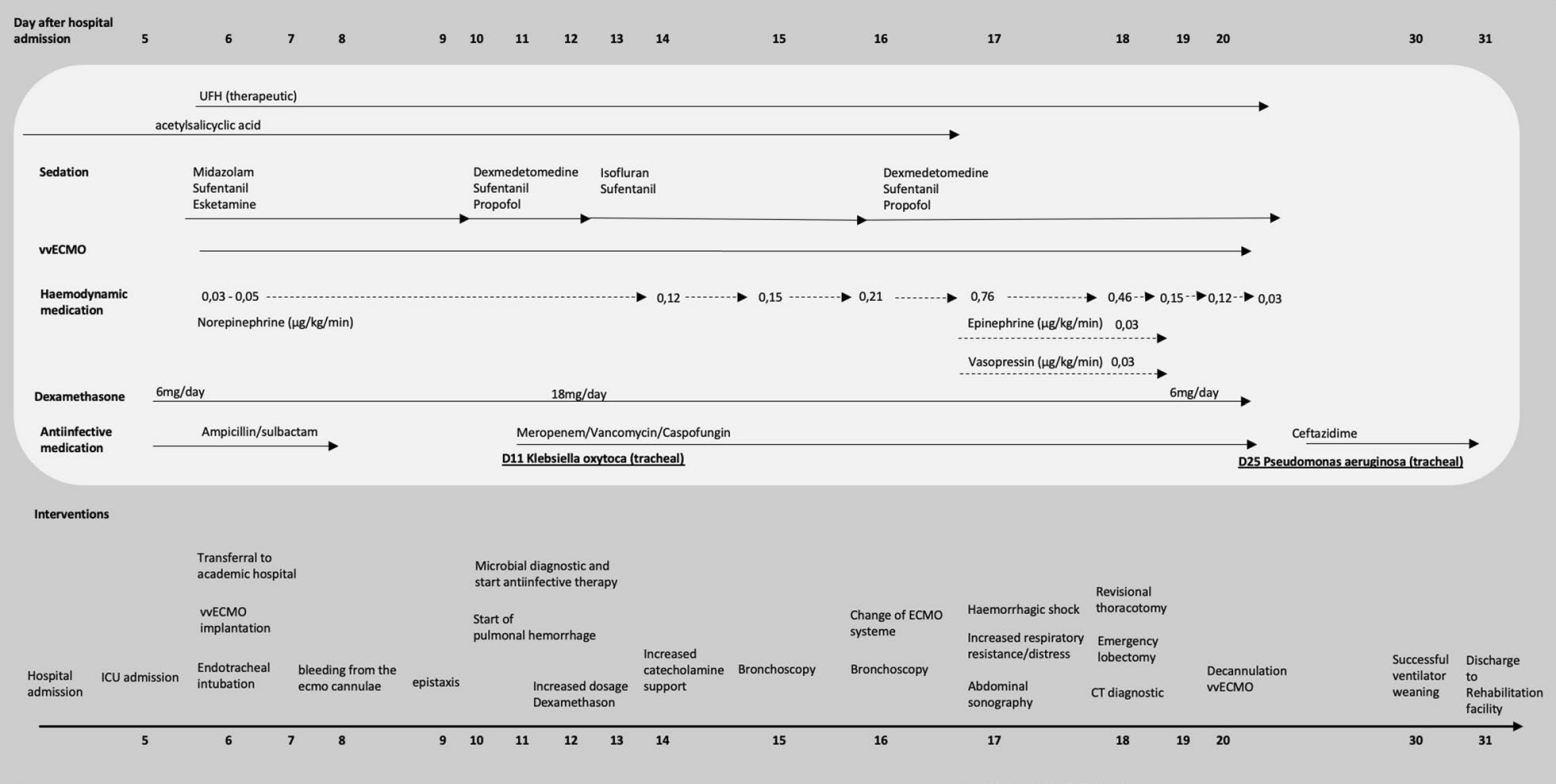
Timeline

He received therapeutic anticoagulation with unfractionated heparin (UFH). Monitoring of anticoagulation was done by measurement of activated partial thromboplastin time (aPTT) every 6 hours with a target of 50–60 seconds. Preexisting treatment with acetylsalicylic acid was continued. Sedation proved to be difficult even under a multimodal intravenous sedative regime. First signs of pulmonary hemorrhage were noticed on day 11. Because of a suspected hyperinflammatory syndrome [4], dosage of dexamethasone was increased from 6 to 18 mg per day. For a detailed depiction of events, see timeline (Fig. 1 and Additional file 1).

**Fig. 2:**
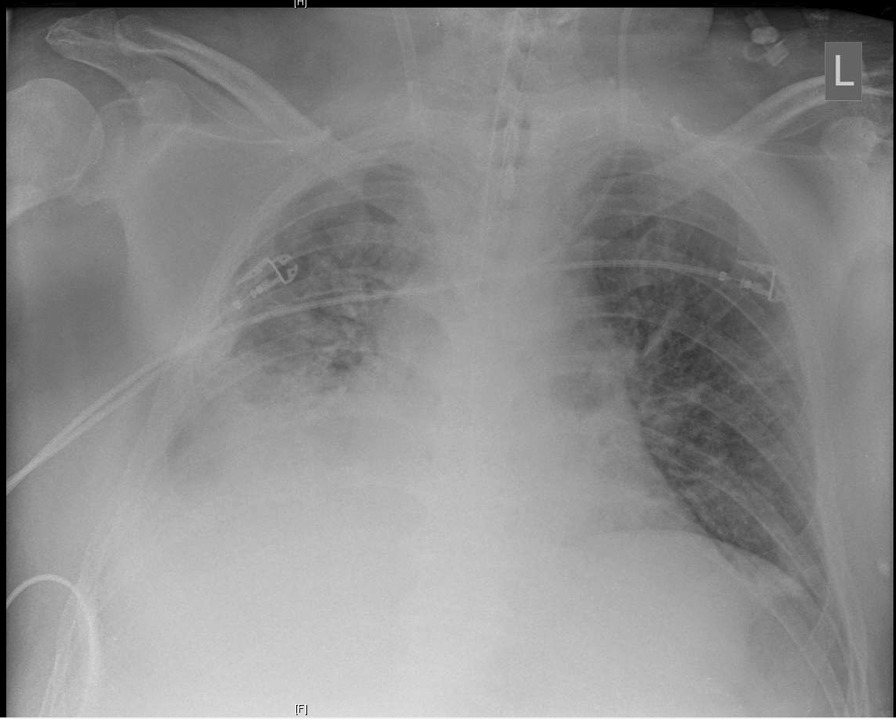
Chest x-ray on day 15

Over the following days, the patient continuously produced extensive aqueous and slightly bloody discolored tracheal secretion. Chest x-ray on day 15 in supine position showed a progressive extensive opacity projecting to the right inferior lobe (Fig. 2). This corresponded with a heavy decline in pulmonary gas exchange. A diagnostic bronchoscopy on the same day revealed hemorrhagic pneumonia with extremely vulnerable mucosa and plenty of viscous mucus as well as extensive coagulum in segment 9. After removal of the coagulum, gas exchange improved at first. Because pulmonary hemorrhage remained persistent and gas exchange declined yet again alarmingly, the patient underwent a second bronchoscopy on the following day. Here, another extensive coagulum in right lung segment nine was cautiously and, due to high risk of bleeding, incompletely recovered. Dependence of extracorporeal support remained very high, and the patient became increasingly unstable. On day 17, the patient developed profound hemorrhagic shock (Table 1) without evident source of bleeding. The patient required extensive doses of norepinephrine, epinephrine, and vasopressin as well as a massive transfusion of red blood cells, plasma, and platelets for stabilization. At the same time, a severe acute decrease of pulmonary compliance was noticed.

**Table 1:** Blood gas analysis over the days of hospitalization

	Day 6 (after ECMO implantation)	Day 17 (occult shock)	Day 18 (time of CT diagnostic)	Day 18 (thoracotomy)	Day 18 (revisional surgery)	Day 19 (first postoperative day)	Day 31 (referral to rehabilitation clinic)
FiO_2_%	100%	100%	100%	100%	100%	30%	2 LPM
pH	7.47	7.22	7.42	7.17	7.19	7.48	7.49
SpO_2_ (%)	97	96	97	96	90	95	92
PaO_2_ (mmHg)	88	101	89	107	67	77	64
PaCO_2_ (mmHg)	29	55	41	76	67	39	41
Hemoglobin (g/dl)	12.7	5.3	11.2	13.9	6.7	9.8	10.2
Potassium (mmol/l)	3.9	5.8	5.7	6.5	6.1	3.7	3.5
Lactate (mmol/l)	1.2	3.7	1.7	1.8	3.7	1.4	0.6
Base excess (mmol/l)	1.3	−5.7	1.9	−3.8	−3.0	4.4	7.4

*FiO*_*2*_ fraction of inspired oxygen, *SpO*_*2*_ oxygen saturation at periphery, *PaO*_*2*_ partial pressure of oxygen, *PaCO*_*2*_ partial pressure of carbon dioxide, *LPM* liters of oxygen per minute.

## Diagnostic focus and assessment/investigations

**Fig. 3:**
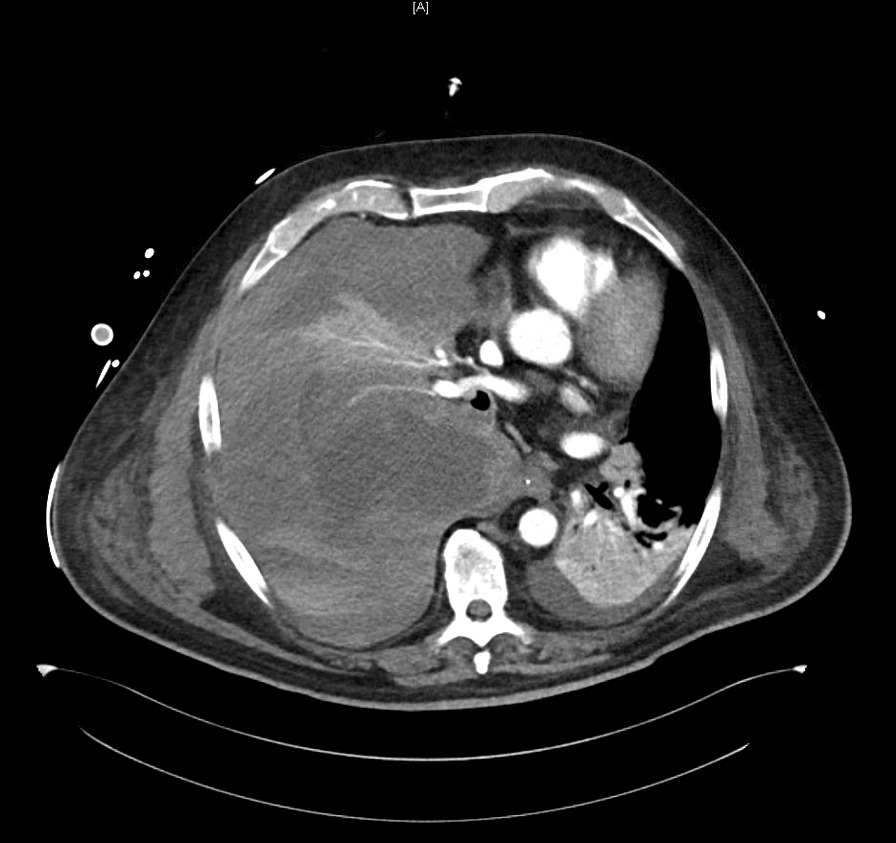
Axial slice from arterial phase computed tomography (Se:8, lm:59)

**Fig. 4:**
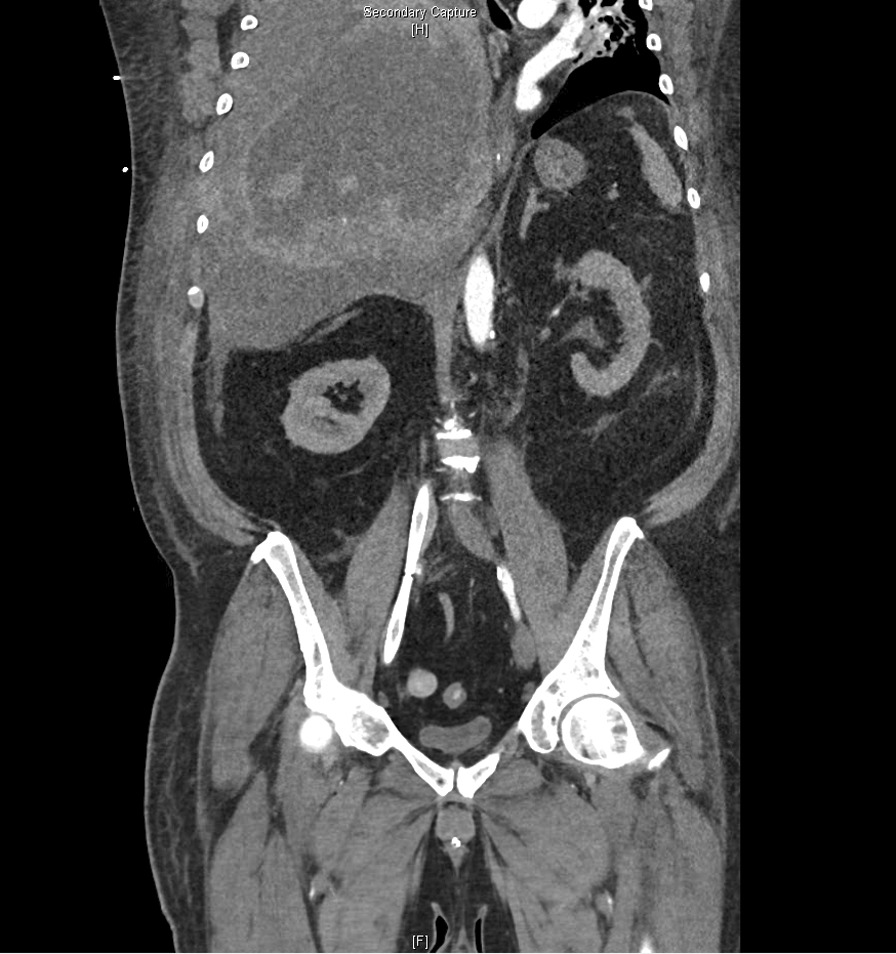
Coronal slice from multiplanar reformation computed tomography (Se:904, lm82). Phrenoptosis due to massive hemothorax of the right hemithorax

Since the patient showed multiple bleeding complications, we suspected an ECMO-associated coagulopathy. Acquired von Willebrand syndrome was ruled out (vWF-activity 246%, vWF antigen 441%), as well as a clotting factor deficiency (factors II 107%, V > 180%, XIII 64.1%). Point-of-care coagulation diagnostic on day 13 showed no signs of fibrinolysis but displayed a rather hypercoagulable state (see Additional file 2) despite anticoagulant therapy with unfractionated heparin and acetylsalicylic acid. The patient had a normal platelet count until the day he developed hemorrhagic shock. At the time of the massive bleeding event, aPTT ranged between 38 and 50 seconds. Occurrence of bloody discolored tracheal secretion was thought to be the result of a possible aspiration event of blood in the hypopharynx due to nasal bleeding. Since the bloody discolored tracheal secretion remained persistent and was of very aqueous consistence, the working diagnosis changed to lung edema. The patient required large amounts of sedatives and was repeatedly disharmonic to the respirator, so that we suspected a self-inflicted lung injury as causal to the potential lung edema. On day 18, when the patient was in manifest shock, serum transaminases were severely elevated, indicative of a severe liver damage. Renal parameters displayed acute kidney injury KDIGO stage 2. Focused assessment with sonography revealed a large pleural effusion in the right hemithorax; transthoracic echocardiography led to the conclusion of hypovolemic shock. After stabilization of the patient, a CT scan of head, thorax, abdomen, and pelvis was done, revealing a massive hemothorax in the right hemithorax with complete compression of the ipsilateral lung, mediastinal shift, and signs of acute bleeding from a pulmonary artery (Fig. 3). There were also extensive low-contrast lesions in liver segments 6, 7, and 4a, indicative of low perfusion of these segments due to compression of the liver parenchyma in effusion-associated phrenoptosis at the right side (Fig. 4). On thoracic CT angiography, no signs of pulmonal arterial aneurysm were seen.

## Therapeutic focus and assessment

After CT scan, a posterolateral thoracotomy through the fifth intercostal space with evacuation of a massive hematoma of approximately 4–5 L blood was executed. The right inferior lobe was confirmed as the source of the bleeding. It presented itself imbibed and starkly altered and had to be resected to stop the bleeding. After lobectomy, the patient stabilized temporarily. A few hours after initial surgery, a second hemorrhagic shock due to a massive intrathoracic bleeding occurred. Revisional surgery revealed another massive hemothorax supplied by another approximate 5 L of blood originating from an intercostal artery located in the intercostal space below the recent thoracotomy.

According to the pathology report of the removed lung tissue, the dorsal inferior lobe presented itself with extensive hemorrhage in an area of 10 × 12 cm. In bled-in areas, there was subpleural focal necrosis with a maximum diameter of 2 cm.

**Fig. 5:**
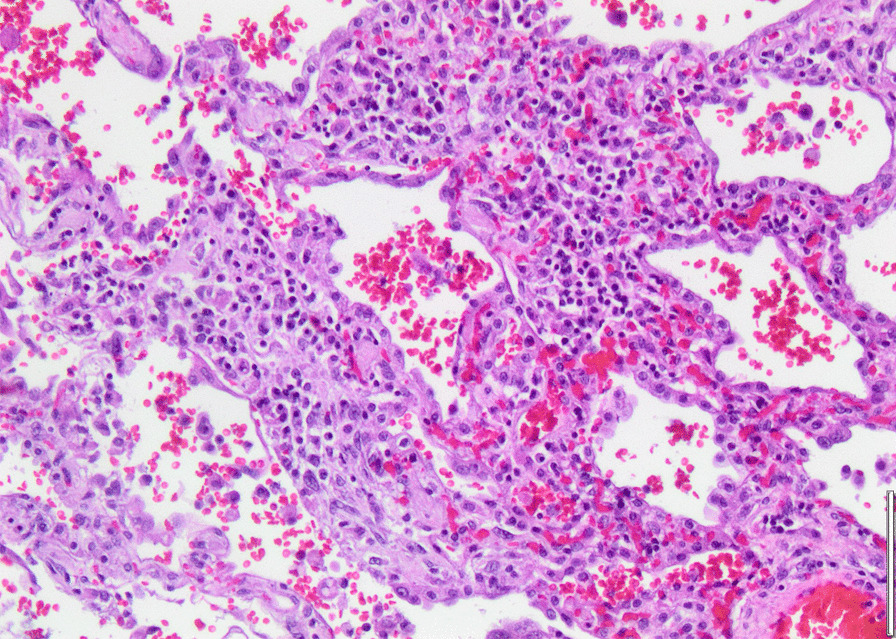
Pneumonitis

**Fig. 6:**
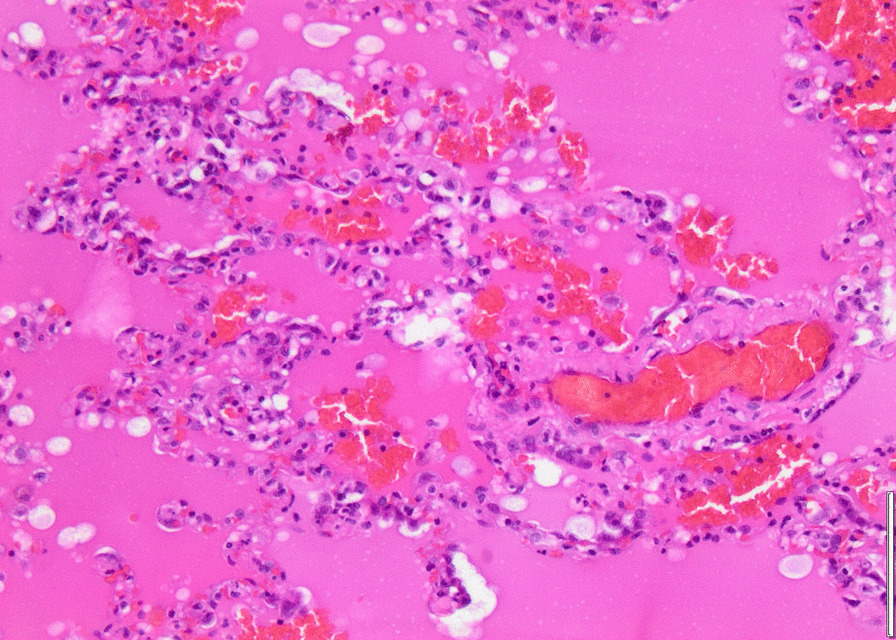
Edema

**Fig. 7:**
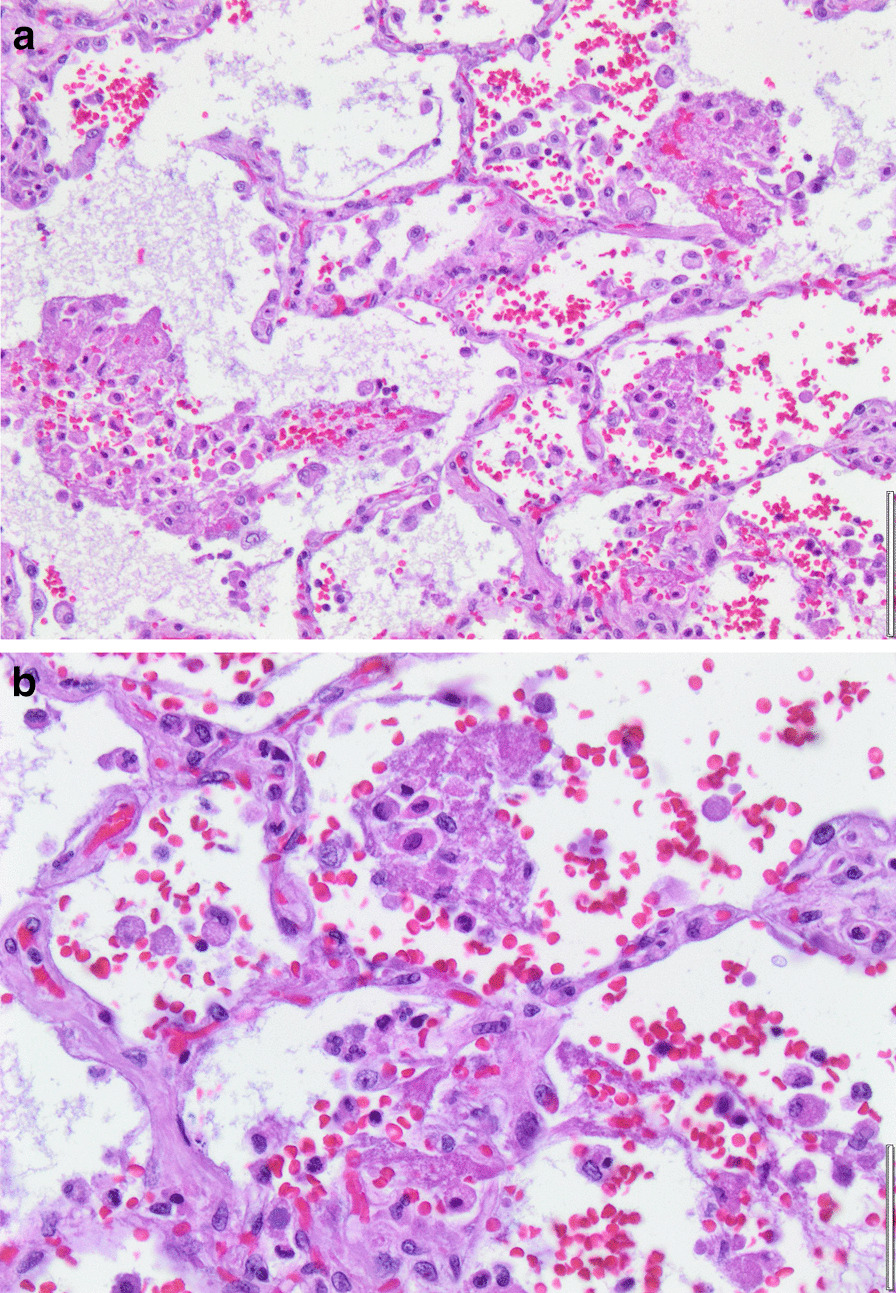
**a** Fibrinous pneumonia. **b** Fibrinous pneumonia

**Fig. 8:**
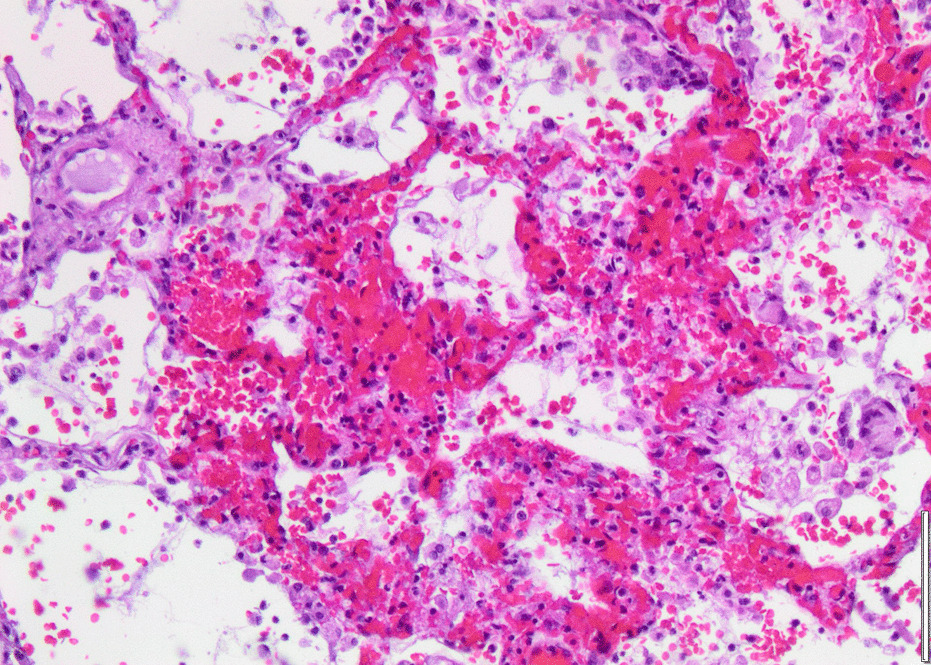
Necrosis

**Fig. 9:**
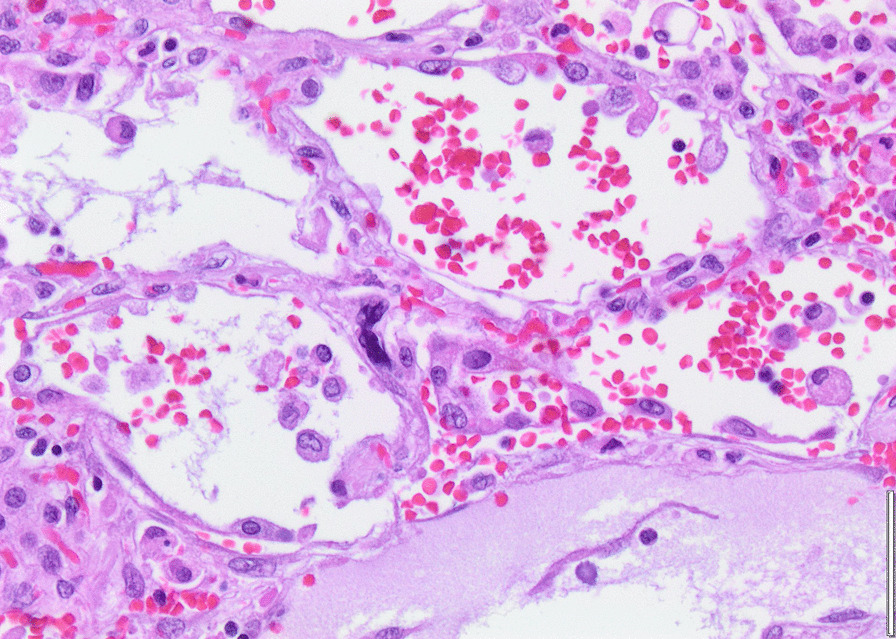
Intracapillary megakaryocyte

**Fig. 10:**
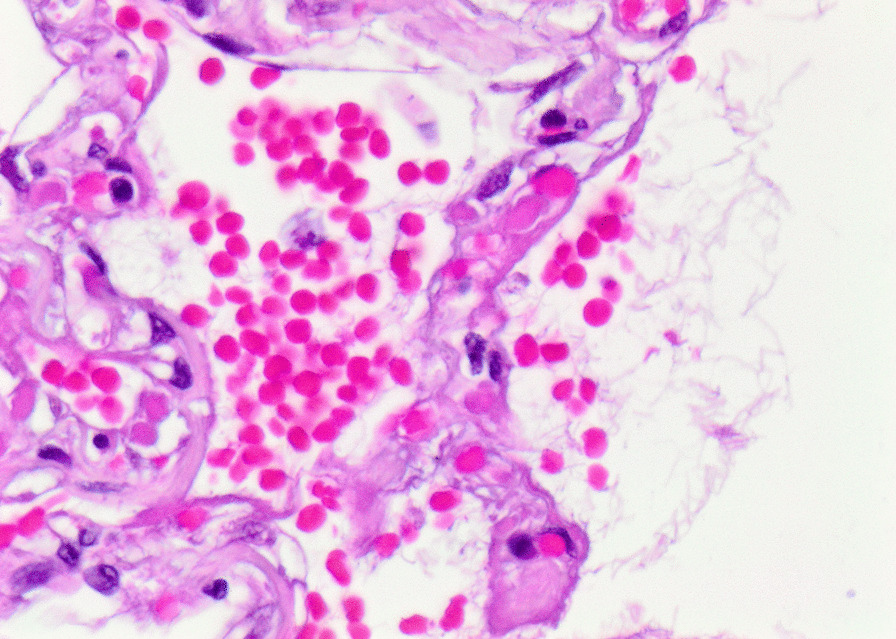
Intracapillary thrombi

Histopathology revealed an organizing fibrinous focal pneumonia (Figs. 5, 6, 7a and b) with focal parenchymal necrosis (Fig. 8) and extensive hemorrhage of lung parenchyma as well as multifocal alveolar capillary microthrombi (Figs. 9 and 10). The morphological finding was seen to be in accordance with the pattern of acute viral pneumonia.

## Outcome and follow-up

After emergency lobectomy of the right inferior lobe, evacuation of the hemothorax, and sufficient hemostasis, the patient underwent a fast recovery. ECMO support was terminated 2 days after lobectomy. The patient was able to breath constantly without respirator support on postoperative day 11. A tracheal culture taken on day 25 revealed bacterial superinfection with *Pseudomonas aeruginosa*, which was treated with ceftazidime. After cessation of sedation, ICU-acquired weakness with proximal flaccid tetraparesis became evident. He was discharged to rehabilitation 31 days after initial hospital admission.

## Discussion

### Brief review of the literature

This is, to the best of our knowledge, the second reported case of a spontaneous intrathoracic bleeding in a patient with COVID-19 [5]. In both cases, evidence of parenchymal necrosis of the lungs as a complication of the SARS-CoV-2-induced pneumonia was found. This pattern is in accordance with past findings that the occurrence of hemoptysis in nontuberculous lower respiratory tract infections is strongly associated with necrotizing pneumonia [2]. Pulmonary hemorrhage seems to be a rare but still recurring complication of SARS-CoV-2-infection. In a systematic review, 22% of ante- or postmortem dissected lungs displayed macroscopic hemorrhagic changes. Histopathologically, alveolar hemorrhage was seen in 33% and partial hemorrhagic necrosis in 0.3% of cases [6]. Clinically evident pulmonary hemorrhage has been described in patients with SARS-CoV-2-associated pneumonia with and without therapeutic anticoagulation [7, 8]. In COVID-19 patients with severe ARDS and therapeutic anticoagulation under vvECMO therapy, incidence of pulmonary hemorrhage seems to occur in a substantial number of cases. In a monocentric observation study, 17% of COVID-19 patients with ECMO support developed pulmonary hemorrhage that required intervention. Three cases were successfully controlled by temporary disruption of heparin for few days, and in one case successful embolization of a pulmonary artery was performed [1]. Murgo *et al.* reported another case of severe pulmonary hemorrhage in a patient with COVID-19 on ECMO therapy. Seven days after initiation of vvECMO therapy, the patient developed profound pulmonary bleeding. Several bronchoscopic interventions were required because of recurrent formation of obstructive clots. Since the endobronchial bleeding kept recurring and the patient was in a critical condition, complete bilateral embolization was performed as *ultima ratio*. Endobronchial bleeding stopped after the procedure, but the patient died nonetheless from nonhemorrhagic shock 3 days after the embolization [9]. Goursaud *et al.* reported a case of necrotizing pneumonia with spontaneous hemothorax in a patient with COVID-19 under ECMO support. After management of the bleeding complication with a thoracic drainage and massive transfusion, the patient died from refractory vasoplegic shock related to a massive systemic inflammatory response syndrome [5]. Necrotizing pneumonia is a rare complication in lung parenchyma infections and is defined by the development of parenchymal necrosis. Risk factors for the development of necrotizing pneumonia involve the inflammatory response of the host and thrombosis of the pulmonary vasculature. A further risk factor is coinfection of the lungs with bacterial and viral pathogens [3]. Necrotizing pneumonia is usually associated with bacterial infections, especially with *Klebsiella* species, *Streptococcus pneumoniae*, and *Staphylococcus aureus* [13]. Rapidly progressing necrotizing pneumonia has been reported in patients with bacterially superinfected influenza pneumonia [3]. In patients with necrotizing pneumonia, affection of pulmonal artery vasculature was seen in about 40% of the cases [2]. Considering the high prevalence of capillary microthrombosis and high inflammatory burden as well as a significant rate of bacterial coinfection in patients suffering from severe COVID-19, the ideal basis for necrotizing pneumonia is given. It still seems to be a rare complication that develops only in the most affected patients. In cases of necrotizing pneumonia, profound parenchymal injury and necrosis can lead to complications such as spontaneous pneumothorax or erosion of pulmonal vasculature resulting in pulmonary bleeding, or, as in our case, even in intrathoracic bleeding.

### Case discussion

Pathophysiology of SARS-CoV-2-induced lung damage is still incompletely understood. There is, however, accumulating evidence of profound damage to the lung parenchyma in severe cases [10–12]. In our patient, there was no mechanical trauma that could explain the occurrence of the pulmonal vascular injury leading to the massive intrathoracic bleeding. Intraoperatively, the right inferior lobe stood out as severely altered and imbibed. Histopathologically, the changes of the tissue were consistent with those of an acute viral pneumonia in the organizing phase with focal parenchymal necrosis.

**Fig. 11:**
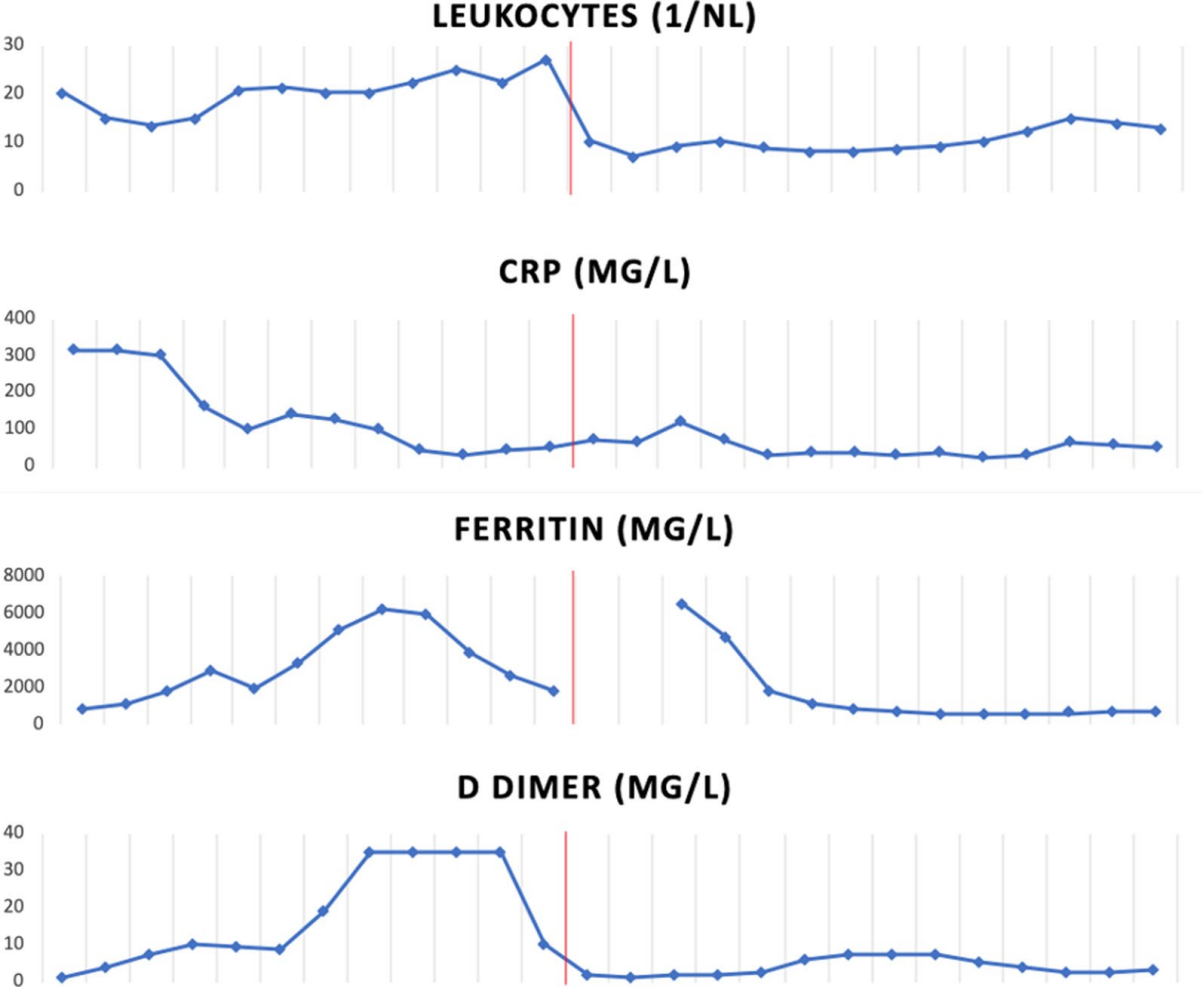
Time course of inflammation markers and d-dimer during the ICU stay. Red line indicates day of emergency right lower lobe lobectomy

It has been described that patients with necrotizing pneumonia show pronouncedly elevated inflammatory markers [13]. In our patient, d-dimer, ferritin, and white blood cell count were rising parallel to the occurrence of bloody tracheal secretions and remained massively elevated in the days before the hemorrhagic shock (Fig. 11). The massive elevation of d-dimer was not associated with clinically or radiologically evident thrombosis. Nevertheless, elevated d-dimer, ferritin, and inflammation markers are not specific for a distinct complication but are known to be associated with a poor prognosis in COVID-19 generally [14, 15]. The exact biological mechanism of the markedly elevated d-dimer and the interindividual variations in patients with COVID-19 remain unclear [14]. After resection of the right inferior lobe in our patient, d-dimer and white blood cell count fell instantly and remained only slightly elevated henceforth. Severely elevated inflammatory markers in combination with occurrence of pulmonary hemorrhage in a critically ill COVID-19 patient should alert to the possible development of an otherwise rare necrotizing pneumonia.

Therapeutic approach to necrotizing pneumonia consists of mainly conservative anti-infective and symptomatic treatment. Surgical interventions are reserved for cases not responsive to medical treatment or in case of severe bleeding complications that cannot be controlled by embolization of the affected vessels.

Some aspects of our case management can be discussed. Even though we saw bleeding complications several days before the arterial bleeding, we did not deescalate our anticoagulation regime. In the face of the persistent hypercoagulability even under high UFH dosage and a consecutive oxygenator thrombosis with the necessity of an ECMO system change on day 16, we weighed the risk of a life-threatening thrombotic event higher than that of persistent mild bleeding. When it came to the occult hemorrhagic shock, we reduced UFH dosage to 4 units/kg/hour. Retrospectively, earlier thoracic computed tomographic imaging could have revealed the necrotic conversion of the right inferior lobe before it became evident by arrosion of the pulmonal artery.

Regarding the extent of the hemothorax and computed tomographic signs of active arterial bleeding, we primarily performed open thoracotomy to evaluate the source of the bleeding. Resection of the severely altered right inferior lobe led to a rapid stabilization not only of the hemodynamics, but also of the respiratory situation. After days of persistent complete dependence from the extracorporeal support due to a profound hypoxemia, the patient underwent a surprisingly rapid ECMO weaning and was decannulated 2 days postoperatively (Table 1). This case demonstrates the pathophysiological principle that impaired oxygenation in ARDS is not per se caused by impaired diffusion across the alveolar–capillary membrane but is predominantly caused by right-to-left shunting due to regions where perfusion exceeds ventilation [16]. The fast sequence from high ECMO dependence to ECMO weaning only shortly after the surgical intervention leads to the conclusion that the necrotic right inferior lobe was responsible for a significant amount of right-to-left shunting in this patient. A similar observation was made by Ashkenazi *et al.*, who reported a lobectomy in a toddler on vvECMO therapy due to necrotizing pneumonia without clinical improvement under conservative management. After resection of the problematic lobe, the toddler could be weaned off vvECMO support immediately and was discharged home 2 weeks later [17]. In a monocentric observation study, all patients that underwent surgery for therapy-refractory pulmonary bleeding in necrotizing pneumonia survived and were discharged alive from the ICU [2].

## Conclusion

The combination of severely elevated inflammatory markers and pulmonary hemorrhage should arouse suspicion of necrotizing pneumonia and lead to timely diagnostic. In COVID-19 cases with progression to necrotizing pneumonia that do not respond well to conventional therapy, surgical intervention is an interesting option that warrants further exploration.

## Patient’s perspective

“I fully recovered and now am able to live the life I had before the infection, including sportive activity and being able to take care of my dog. The most persistent problem turned out to be a hypoglossal nerve palsy, which eventually resolved almost completely under speech therapy. I am very thankful to the intensive care team for not giving up on me.”

## Supplementary Information

**Additional file 1.** Detailed timeline of events and medication taken during the stay.

**Additional file 2.** Viscelastometic point-of-care diagnostic (Haemonetics ClotProⓇ) on day 13.

## Data Availability

All data generated or analyzed during this case report are included in this published article.

## Abbreviations

ARDS: Acute respiratory distress syndrome
aPTT: Activated partial thromboplastin time
BMI: Body mass index
COVID-19: Coronavirus disease 2019
CRP: C-reactive protein
CT: Computed tomography
FiO_2_: Fraction of inspired oxygen
ICU: Intensive care unit
IL: Interleukin
KDIGO: Kidney Disease: Improving Global Outcomes
LPM: Liters oxygen per minute
MPR: Multiplanar reformation
PaCO_2_: Partial pressure of carbon dioxide
PaO_2_: Partial pressure of oxygen
PCR: Polymerase chain reaction
SARS-CoV-2: Severe acute respiratory syndrome coronavirus type 2
SpO_2_: Oxygen saturation at periphery
UFH: Unfractionated heparin
vvECMO: Venovenous extracorporeal membrane oxygenation
vWF: von Willebrand factor
