# Supplementary material for: Spontaneous massive hemothorax as a complication of necrotizing pneumonia in a patient with severe acute respiratory syndrome coronavirus 2 induced acute respiratory distress syndrome: a case report
Source: J Med Case Rep. 2021 Sep 3;15:444. doi: 10.1186/s13256-021-03032-9 (PMC8415192; doi:10.1186/s13256-021-03032-9)
Supplement: Supplementary file 1 — Additional file 1. Detailed timeline of events and medication taken during the stay. [file 13256_2021_3032_MOESM1_ESM.pptx]

## Slide 1
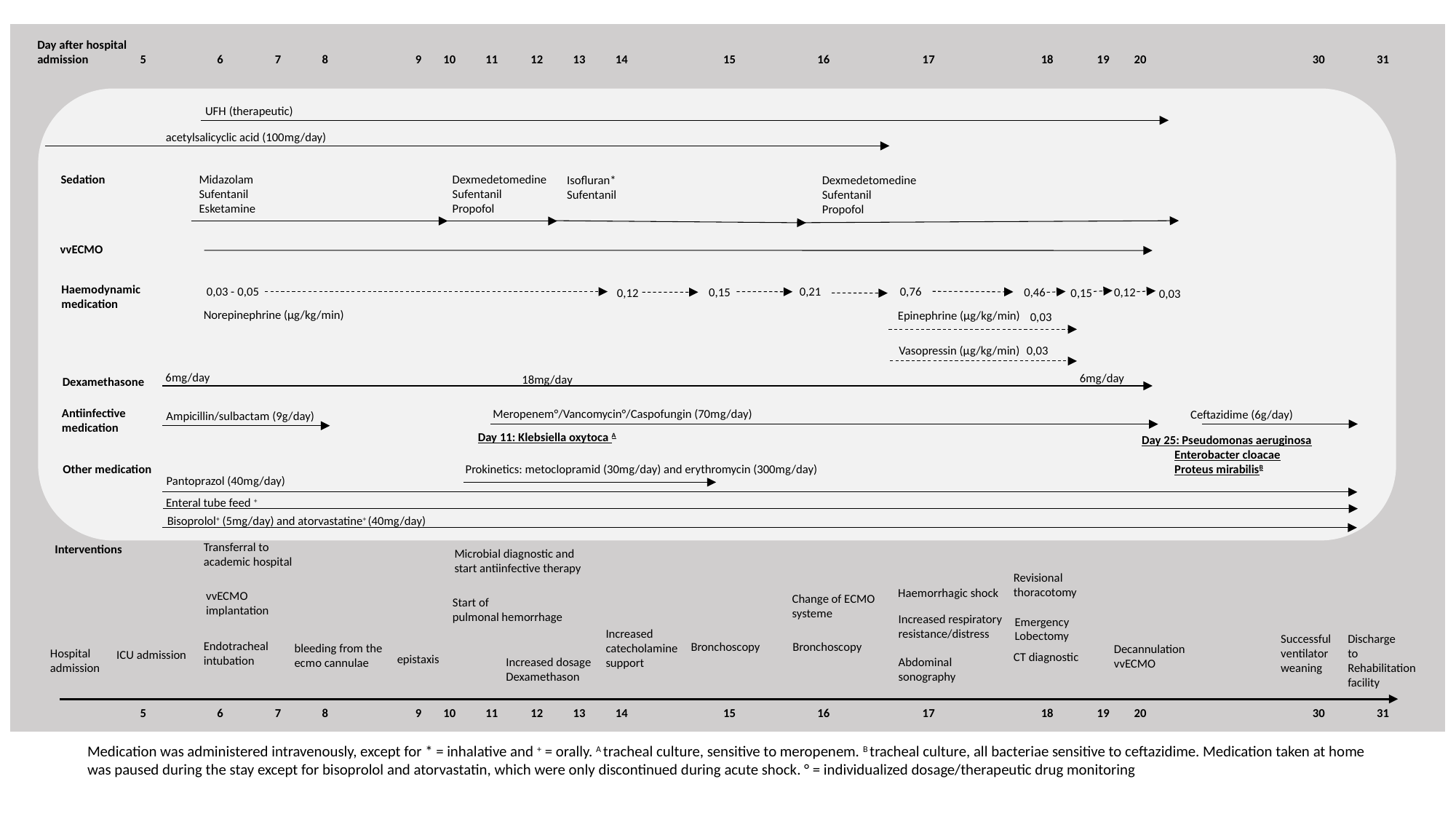

Day after hospital admission
 5 6 7 8 9 10 11 12 13 14 15 16 17 18 19 20 30 31
UFH (therapeutic)
acetylsalicyclic acid (100mg/day)
Dexmedetomedine
Sufentanil
Propofol
Midazolam
Sufentanil
Esketamine
Sedation
Dexmedetomedine
Sufentanil
Propofol
Isofluran*
Sufentanil
vvECMO
Haemodynamic
medication
0,21
0,03 - 0,05
0,76
0,46
0,15
0,12
0,15
0,12
0,03
Norepinephrine (µg/kg/min)
Epinephrine (µg/kg/min)
0,03
0,03
Vasopressin (µg/kg/min)
6mg/day
6mg/day
18mg/day
Dexamethasone
Antiinfective
medication
Meropenem°/Vancomycin°/Caspofungin (70mg/day)
Ceftazidime (6g/day)
Ampicillin/sulbactam (9g/day)
Day 11: Klebsiella oxytoca A
Day 25: Pseudomonas aeruginosa
 Enterobacter cloacae
 Proteus mirabilisB
Other medication
Prokinetics: metoclopramid (30mg/day) and erythromycin (300mg/day)
Pantoprazol (40mg/day)
Enteral tube feed +
Bisoprolol+ (5mg/day) and atorvastatine+ (40mg/day)
Transferral to
academic hospital
Interventions
Microbial diagnostic and
start antiinfective therapy
Revisional
thoracotomy
Haemorrhagic shock
vvECMO
implantation
Change of ECMO
systeme
Start of
pulmonal hemorrhage
Increased respiratory
resistance/distress
Emergency
Lobectomy
Increased
catecholamine
support
Successful
ventilator
weaning
Discharge
to
Rehabilitation
facility
Endotracheal
intubation
Bronchoscopy
Bronchoscopy
bleeding from the
ecmo cannulae
Decannulation
vvECMO
Hospital
admission
ICU admission
CT diagnostic
epistaxis
Increased dosage
Dexamethason
Abdominal
sonography
 5 6 7 8 9 10 11 12 13 14 15 16 17 18 19 20 30 31
Medication was administered intravenously, except for * = inhalative and + = orally. A tracheal culture, sensitive to meropenem. B tracheal culture, all bacteriae sensitive to ceftazidime. Medication taken at home
was paused during the stay except for bisoprolol and atorvastatin, which were only discontinued during acute shock. ° = individualized dosage/therapeutic drug monitoring
