# Supplementary material for: Spontaneous massive hemothorax as a complication of necrotizing pneumonia in a patient with severe acute respiratory syndrome coronavirus 2 induced acute respiratory distress syndrome: a case report
Source: J Med Case Rep. 2021 Sep 3;15:444. doi: 10.1186/s13256-021-03032-9 (PMC8415192; doi:10.1186/s13256-021-03032-9)
Supplement: Supplementary file 2 — Additional file 2. Viscelastometic point-of-care diagnostic (Haemonetics ClotProⓇ) on day 13. [file 13256_2021_3032_MOESM2_ESM.pptx]

## Slide 1
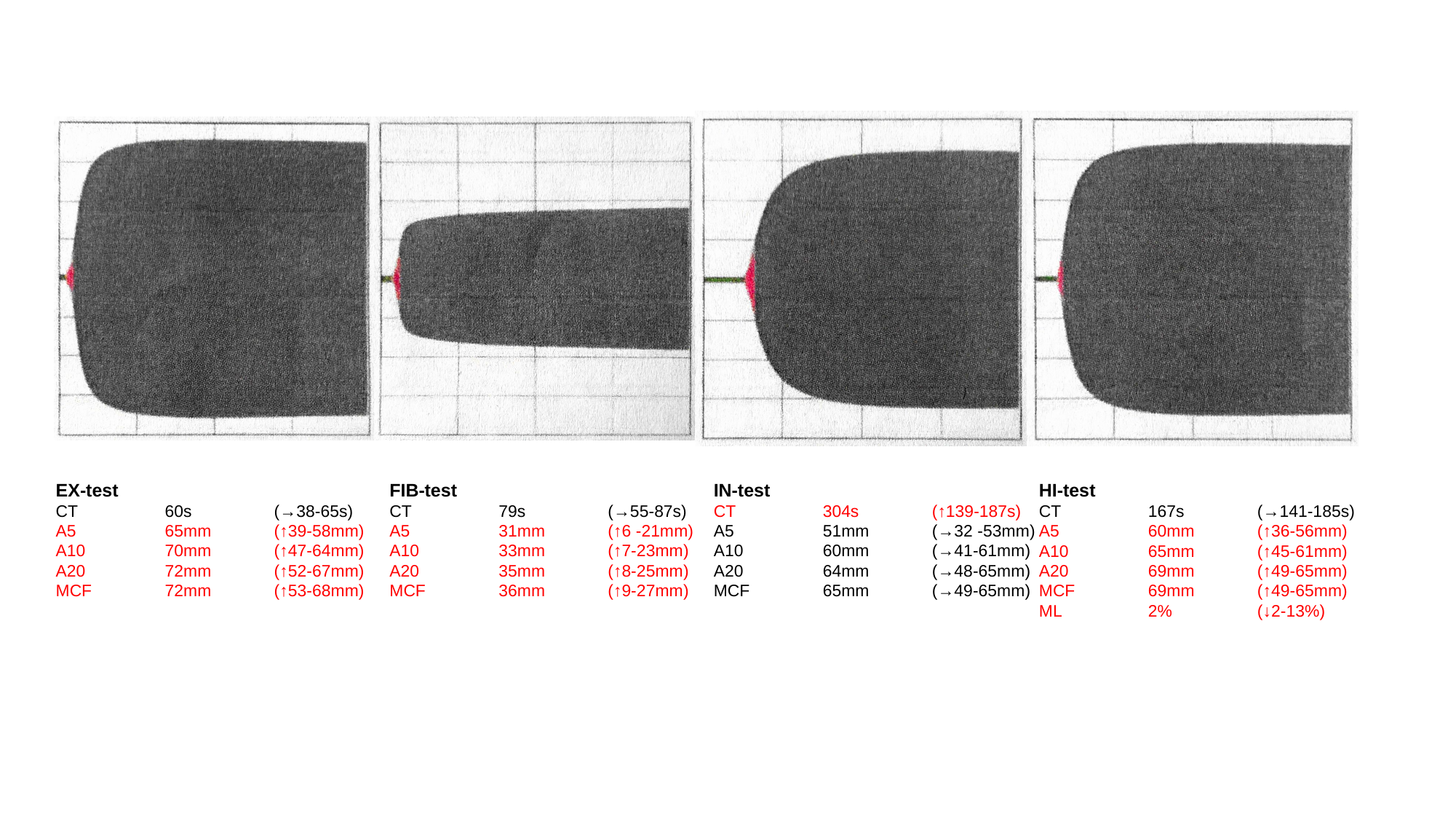

EX-test
CT 	60s 	(→38-65s)
A5 	65mm 	(↑39-58mm)
A10 	70mm 	(↑47-64mm)
A20 	72mm 	(↑52-67mm)
MCF 	72mm 	(↑53-68mm)
FIB-test
CT 	79s 	(→55-87s)
A5 	31mm 	(↑6 -21mm)
A10 	33mm 	(↑7-23mm)
A20 	35mm 	(↑8-25mm)
MCF 	36mm 	(↑9-27mm)
IN-test
CT 	304s 	(↑139-187s)
A5 	51mm 	(→32 -53mm)
A10 	60mm 	(→41-61mm)
A20 	64mm 	(→48-65mm)
MCF 	65mm 	(→49-65mm)
HI-test
CT 	167s 	(→141-185s)
A5 	60mm 	(↑36-56mm)
A10 	65mm 	(↑45-61mm)
A20 	69mm 	(↑49-65mm)
MCF 	69mm 	(↑49-65mm)
ML 	2% 	(↓2-13%)
